# Sulfoxaflor exposure reduces egg laying in bumblebees *Bombus terrestris*

**DOI:** 10.1111/1365-2664.13519

**Published:** 2019-10-29

**Authors:** Harry Siviter, Jacob Horner, Mark J. F. Brown, Ellouise Leadbeater

**Affiliations:** ^1^ Department of Biological Sciences Royal Holloway University of London Egham UK

**Keywords:** bee, insecticide, neonicotinoid, ovary development, pesticide, reproductive output, sucrose consumption, sulfoximine

## Abstract

1. Sulfoximine‐based insecticides, such as sulfoxaflor, are of increasing global importance and have been registered for use in 81 countries, offering a potential alternative to neonicotinoid insecticides.
2. Previous studies have demonstrated that sulfoxaflor exposure can have a negative impact on the reproductive output of bumblebee colonies, but the specific life‐history variables that underlie these effects remain unknown.
3. Here, we used a microcolony‐based protocol to assess the sub‐lethal effects of chronic sulfoxaflor exposure on egg laying, larval production, ovary development, sucrose consumption, and mortality in bumblebees. Following a pre‐registered design, we exposed colonies to sucrose solutions containing 0, 5, 10 and 250ppb of sulfoxaflor. Exposure at 5 ppb has been previously shown to negatively impact colony reproductive success.
4. Our results showed that sulfoxaflor exposure at 5 ppb (lowest exposure tested) reduced the number of eggs found within the microcolonies (Hedge's *d* = −0.37), with exposed microcolonies also less likely to produce larvae (Hedge's *d* = −0.36). Despite this, we found no effect of sulfoxaflor exposure on ovarian development. Sulfoxaflor‐exposed bumblebees consumed less sucrose solution, potentially driving the observed reduction in egg laying.
5. *Policy implications*. Regulatory bodies such as the European Food Safety Authority (EFSA) are under increasing pressure to consider the potential impact of insecticides on wild bees, such as bumblebees, but sublethal effects can go undetected at lower‐tier testing. In identifying just such an effect for bumblebees exposed to sulfoxaflor, this study highlights that microcolony‐based protocols are a useful tool that could be implemented within an ecotoxicology framework. Furthermore, the results provide evidence for potentially negative consequences of pollinator exposure to an insecticide that is currently undergoing the licensing process in several EU member states.

## Graphical abstract

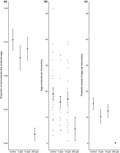

## 1 INTRODUCTION

Neonicotinoids are the most commonly used insecticides worldwide (Simon‐Delso et al., 2015), but evidence demonstrating their negative sub‐lethal impacts on important pollinators, such as bees (Rundlöf et al., 2015; Siviter, Koricheva, Brown, & Leadbeater, 2018; Tsvetkov et al., 2017; Woodcock et al., 2017), has resulted in legislative reassessment globally. Most noticeably, within the European Union, three commonly used neonicotinoids (thiamethoxam, imidacloprid, and clothianidin) are now banned from agricultural use outside of commercial greenhouses. In contrast to neonicotinoids, sulfoxaflor, the first branded sulfoximine‐based insecticide, is an increasingly important insecticide product that is now registered for use in 81 countries, offering an alternative to neonicotinoid‐based insecticides (Brown et al., 2016). However, the legislative reassessment of neonicotinoid‐based insecticides was driven by research that demonstrated the potential sub‐lethal consequences of neonicotinoid exposure on pollinators (European Commission, 2018). The regulatory process by which novel agrochemicals are licensed for use is changing in Europe and North America, but in its current form, is largely reliant on tier‐based toxicity tests that can fail to detect sub‐lethal effects at lower tiers. Therefore, despite sulfoximine‐based insecticides and neonicotinoids having a similar biological mode of action as selective agonists of Nicotinic Acetyl Choline Receptors (NAChRs) (Sparks et al., 2013; Zhu et al., 2011), we still have a limited understanding of the potential sub‐lethal effects of sulfoxaflor on bee colonies.

Siviter, Brown, and Leadbeater (2018) recently demonstrated that chronic exposure to sulfoxaflor at a concentration of 5ppb had negative consequences for the worker production and reproductive output of bumblebee *(Bombus terrestris audax*) colonies. Colony‐level impacts of neonicotinoid exposure on bees are thought to be driven in part by impaired bee foraging behaviour and cognition (Feltham, Park, & Goulson, 2014; Gill, Ramos‐Rodriguez, & Raine, 2012; Klein, Cabirol, Devaud, Barron, & Lihoreau, 2017; Lämsä, Kuusela, Tuomi, Juntunen, & Watts, 2018; Muth & Leonard, 2019; Samuelson, Chen‐Wishart, Gill, & Leadbeater, 2016; Siviter, Koricheva, et al., 2018; Stanley, Smith, & Raine, 2015). Interestingly, in both Siviter, Brown, et al. (2018), and a follow‐up study (Siviter, Scott, et al., 2019), no significant effect of sulfoxaflor exposure on either bumblebee foraging behaviour or cognition was observed, and consequently, the mechanism behind the sub‐lethal colony‐level effects of sulfoxaflor remains unknown. An alternative explanation, again based on previous work with neonicotinoids (Baron, Jansen, Brown, & Raine, 2017; Baron, Raine, & Brown, 2017; Laycock, Lenthall, Barratt, & Cresswell, 2012), is that exposure to sulfoxaflor early in the colony life cycle could reduce egg laying, or impair larval development, with downstream consequences for reproductive output (Siviter, Brown, et al., 2018).

Neonicotinoid insecticides can negatively influence bumblebee ovary development and fecundity (Baron, Raine, et al., 2017; Laycock & Cresswell, 2013; Laycock et al., 2012). For example, Laycock et al. (2012) demonstrated that queenless microcolonies exposed to field realistic concentrations of imidacloprid had a one‐third reduction in the total amount of brood produced. These effects occurred in the absence of impacts on ovary development at field realistic concentrations, with the reduced reproductive output instead most likely mediated through lower feeding rates in exposed microcolonies. Baron, Raine, et al. (2017) showed that exposure to field realistic concentrations of thiamethoxam reduced the average length of terminal oocytes in the ovaries of queens in four wild bumblebee species, (*Bombus lucorum*, *B pascuorum*, *B. pratorum*, *B. terrestris*), with knock‐on consequences for colony initiation and egg laying (Baron, Raine, et al., 2017).

Bumblebee workers are able to produce male offspring if the queen dies or is deposed, and will show signs of ovarian development approximately 7 days after being removed from the colony (Alaux, Boutot, Jaisson, & Hefetz, 2007; Amsalem, Twele, Francke, & Hefetz, 2009). We manipulated this reproductive plasticity and, following Laycock et al. (2012), created queenless microcolonies that were subsequently exposed to varying dosages of sulfoxaflor within sucrose. We monitored the sucrose consumption, mortality and egg laying of bumblebee workers (*Bombus terrestris*) chronically exposed to sulfoxaflor over a 14‐day period. After the 14 days of exposure, we recorded the number of eggs/larvae produced by each microcolony and dissected and measured the ovaries of each surviving worker. Based on Siviter, Brown, et al. (2018), we hypothesized that sulfoxaflor exposure may have a negative impact on bumblebee ovarian development, with knock on effects for egg laying, and larval development.

## 2 MATERIALS AND METHODS

### 2.1 Insecticide exposure

Sulfoxaflor‐based insecticides have been developed for a range of different crops and are most commonly used as a spray application. The residue levels of systemic insecticides vary from crop to crop (Bonmatin et al., 2015; Kyriakopoulou et al., 2017), but despite sulfoxaflor being licenced for use in 81 countries, there is still a limited understanding of the likely post‐spray residue levels that are to be expected in the nectar and pollen of sulfoxaflor‐treated crops. We based our dosages on Environmental Protection Agency (EPA) data that showed that the residue levels of sulfoxaflor range between 5.41 and 46.97ppb in the nectar of sulfoxaflor‐sprayed cotton across a 11‐day period (United States Environmental Protection Agency, 2016; application rate: 0.045 pounds (0.020 kg) of active ingredient per acre applied twice). It is worth noting that in the same EPA study, pollen residue levels were higher than nectar levels (ranging between 50.12 and 510.95ppb; United States Environmental Protection Agency, 2016) and that while spraying bee‐attractive crops during flowering was previously prohibited in North America (EPA, 2019), this is no longer the case, and nor is it the case globally (Dow AgroSciences Australia Limited, 2018; Dow AgroSciences New Zealand, 2018; Dow AgroSciences South Africa, 2018). Our sulfoxaflor treatments were derived from a stock solution of 1 g/dm^3^ sulfoxaflor (Greyhound Chromatography and Allied Chemicals) in acetone, which was combined with sucrose solution (50°Brix) to make four treatment groups: 5 µg/dm^3^ (5ppb), 10 µg/dm^3^ (10ppb) & 250 µg/dm^3^(250ppb; positive control). These were compared to a control solution containing just acetone 0 µg/dm^3^ (0ppb).

### 2.2 Microcolonies

Seven bumblebee colonies of approximately 100 workers were ordered (Biobest, Westerlo, Belgium) and, upon arrival, five workers from each colony were collected and screened for common bee parasites through faecal examination (*Apicystis bombi*, *Crithidia* spp., and *Nosema* spp.) (Rutrecht & Brown, 2009). All colonies were unparasitized and workers were returned to the colony. From these original colonies, we created 120 queenless microcolonies by randomly placing groups of four workers from the same queen‐right colony in small Perspex boxes (67 × 127 × 50 mm; Allied Plastics). The age of individual workers was not known, although there was no difference in the size of workers between different treatment groups (mean size of workers; Control = 5.24 ± 0.34 mm, 5ppb = 5.21 ± 0.42 mm, 10ppb = 5.21 ± 0.42 mm, 250ppb = 5.23 ± 0.40). Each microcolony contained a gravity feeder with an *ad libitum* supply of untreated sucrose solution (50° Brix). Microcolonies were kept in darkness at 26°C and 50%–60% humidity and then left overnight (workers who died overnight were replaced with workers from the same original colony; *N* = 4).

Insecticide exposure began the following day, when the untreated sucrose solution was replaced with weighed sucrose solution (50° Brix) containing either 0, 5, 10 or 250ppb sulfoxaflor according to the randomly assigned treatment group. Workers that died after exposure began were not replaced. Sucrose remaining in the feeder was measured daily, when the bees were first fed and on the following day, to get a recording of daily feeding (OHAUS advanced portable balance scout STX) by a researcher who was blind to treatment. Pollen balls (1.66 g ± *SD* 0.14) were added to the microcolonies on days 1, 4, 8 and 11. Following Siviter, Brown, et al. (2018), pollen balls were only replaced if eggs had not been laid; in cases when eggs had been laid, more pollen was added. Mortality and egg laying were recorded daily via visual inspection. Seven boxes containing just sucrose and no bees were also included as evaporation controls. The experiment ran for a total of 15 days (1‐day pre‐exposure and 14 days of exposure). The total sample size of 120 microcolonies initially contained a total of 480 bees, with 30 microcolonies in each treatment group.

### 2.3 Fecundity and ovary development

At the end of the experiment, individual bees were frozen at −20°C for later dissection. Pollen balls from the nests were also frozen and examined for the presence of eggs and larvae. Pollen balls that contained brood were dissected, and the number of eggs and larvae counted, with a reference photo taken for each microcolony. Bees were dissected in distilled water to remove their ovaries, using a Nikon (SM2800) dissecting microscope. Bumblebee workers have two ovaries, containing four ovarioles, with each ovariole containing several oocytes. Following (Baron, Raine, et al., 2017; Brown, Loosli, & Schmid‐Hempel, 2000; Laycock et al., 2012), we (a) recorded the presence or absence of developed ovarioles and (b) used an ocular graticule to measure the length of each intact terminal oocyte. The mean of all intact oocytes per bee (mean oocyte size per bee), and the largest oocyte length (maximum per bee) were used as our measure of ovarian development/investment. We successfully dissected and examined the ovaries of 373 bees (control = 102; 5ppb = 110; 10ppb = 105; 250ppb = 56). Thorax width was also recorded using digital callipers (Mitutoyo).

### 2.4 Statistical analysis

The statistical analysis described below was pre‐registered prior to the experiment, as was the experimental design (https://aspredicted.org/vw63q.pdf). Any deviations from the pre‐registration document are noted in the text below.

We followed an information theoretic model selection approach. For each analysis, we considered a full model, other models containing the same random factors (specified below) and all subsets of the fixed factors, and a null model containing just the random factors and the intercept. Parameter estimates and confidence intervals are based on model averaging across the 95% confidence set (i.e. the smallest set of models for which the cumulative Akaike weight (*w_i_*) was equal to or greater than 0.95, where models are added to the set in decreasing order of *w_i_*).

We used a hurdle model to analyse both the number of eggs and larvae (analysed separately) produced per microcolony, with treatment included as a fixed factor and colony of origin included as a random factor. Hurdle models handle excess zeros by incorporating two processes: a binomial process that models the occurrence of zero versus non‐zero values, and a truncated count process that only fits positive (i.e. non‐zero) counts. The estimates thus provide two types of information: (a) whether there is variation across treatments in the likelihood of producing eggs/larvae at all (termed zero‐count process; note that a positive parameter estimate here implies that a zero count is more likely) (b) if eggs/larvae are produced, whether there is variation in the number produced (termed positive‐count process).

A mixed effects Cox proportional hazards model was used to analyse latency to lay eggs, with treatment included as a fixed factor and the original colony and microcolony included as random factors.

Our results (see below) showed that multiple bees within each microcolony showed signs of ovarian development, with no obvious dominant individual in most cases, and we therefore conducted our analysis on all bees. Ovarian development (whether a bee had developed ovaries or not) was analysed using a generalized linear mixed effects model (binomial error distribution, link function = "logit")), with treatment and bee size included as fixed factors, and microcolony nested within colony of origin as a random factor (*n.b.* in our pre‐registered document, we stated that we intended to consider the interaction between treatment and bee size, but when included, the model failed to converge). The mean and maximum oocyte length per bee were analysed using linear mixed effect models (poisson error distributions) with treatment, thorax width, and their interaction included as fixed factors and original colony and microcolony included as random factors. As only seven workers from the positive control had developed ovaries, this treatment was excluded from this analysis.

A linear mixed effects model was also used to analyse sucrose consumption per worker ([sucrose consumed per microcolony – evaporation control]/ number of workers in microcolony), which also included treatment, day and their interaction as fixed factors and microcolony (nested within colony of origin) as random factors. In our pre‐registration document, we failed to consider the possibility of spillage, but spillages did occur during the experiment. Thus, we removed all data points where spillages had been recorded by the experimenter (*n* = 64; control *n* = 12, 5ppb *n* = 15, 10ppb *n* = 15, 250ppb *n* = 21) or where apparently negative consumption occurred, implying spillage of the evaporation control (*n* = 19). This left a final sample size of 1,416 for this section of the analysis.

Our analysis included one additional deviation from our pre‐registration document. We observed higher mortality during the experiment than envisaged and thus chose to additionally analyse individual survival data (time‐to‐death). We used a mixed effects Cox proportional hazards model, with treatment, included as a fixed factor, and original colony and microcolony included as random factors. Throughout the analyses, we used the packages Hmisc, lme4, coxme, MuMIn, ggplot2, glmmTMB (Barton, 2016; Bates, Mächler, Bolker, & Walker, 2015; Brooks et al., 2017; Harrell & Dupont, 2018; Therneau, 2018; Wickham, 2009).

## 3 RESULTS

**Figure 1:**
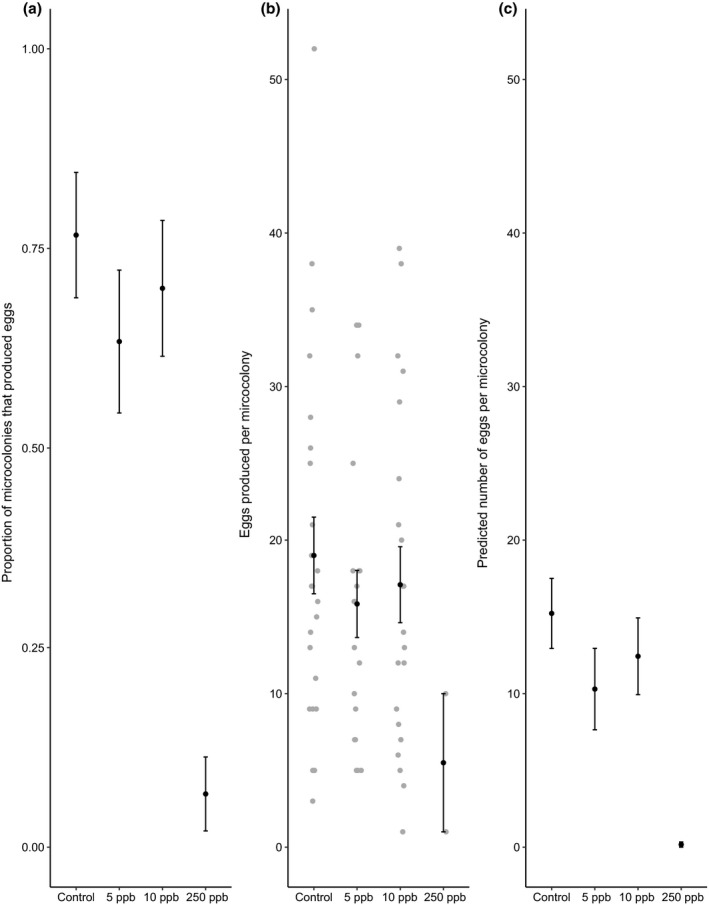
The proportion (±*SE*) of microcolonies that produced eggs (a) and the mean (±*SE*) number of eggs (b) produced per microcolony that produced eggs. The predicted effect (+*SE*) per treatment (c) is derived from the hurdle model and combines the probably of producing eggs and, if eggs were produced, the number produced

Sulfoxaflor exposure did not significantly influence the binary likelihood of microcolonies producing eggs at 5 & 10ppb, although there was an effect at 250ppb, (Figure 1a, hurdle (zero‐count output), 5ppb parameter estimate relative to negative control (PE) = 0.89, 95% confidence intervals (CI) = −0.45 to 2.23; 10ppb PE = 0.47, 95% CI = −0.88 to 1.84, 250ppb PE = 4.87, 95% CI = 2.81 to 6.93). However, we found that in the microcolonies that produced eggs, sulfoxaflor exposure reduced the total number of eggs laid at 5 & 250ppb, although there was no significant difference at 10ppb (Figure 1b, same hurdle model (positive‐count output), 5ppb parameter estimate relative to negative control (PE) = −0.16, 95% CI = −0.31 to −0.01; 10ppb PE = −0.10, 95% CI = −0.24 to 0.04, 250ppb PE = −1.30, 95% CI = −1.91 to −0.70; note however that there was no evidence for an increase between 5ppb and 10ppb; PE (5 vs. 10ppb) = 0.06, 95% CI = −0.09 to 0.21). In other words, for colonies that produced eggs, the number produced was lower than the control for 5ppb and 250ppb, and at 250ppb, more colonies also failed to produce eggs at all.

**Figure 2:**
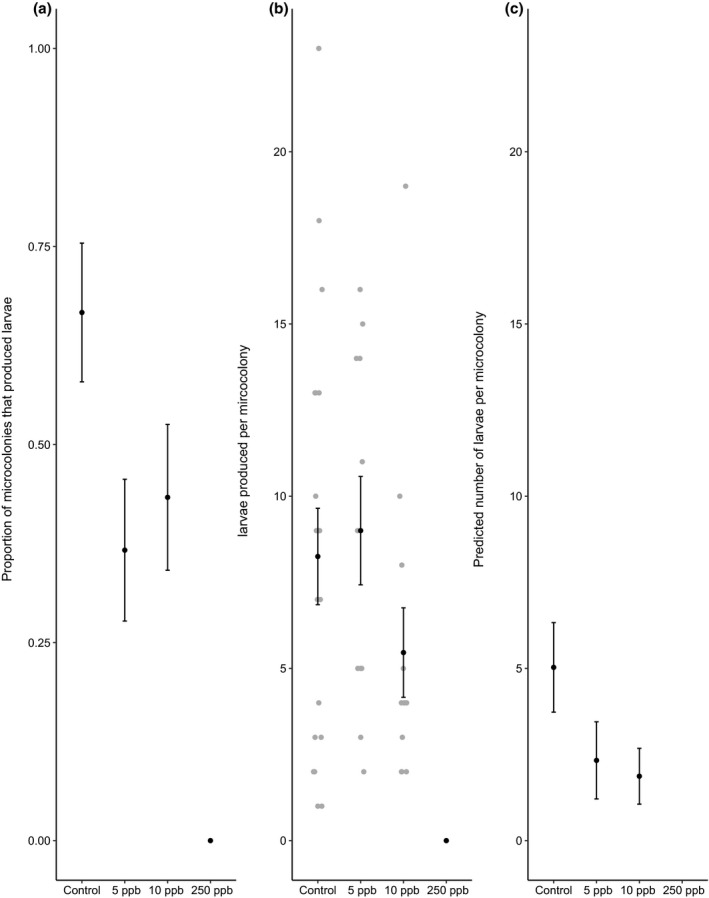
The proportion (±*SE*) of microcolonies that produced larvae (a) and the mean (±*SE*) number of larvae (b) produced per microcolony that produced larvae. The predicted effect (±*SE*) per treatment (c) is derived from the hurdle model and combines the probability of producing larvae and, if larvae were produced, the number produced

We found a similar but more dose‐dependent pattern for the presence of developing larvae (Figure 2). In this case, sulfoxaflor‐exposed microcolonies were less likely than the control to contain any developing larvae at all at 5ppb, but not at 10ppb (Figure 2a, hurdle (zero‐count output), 5ppb, parameter estimate (PE) = 1.24, 95% CI = 0.16 to 2.32; 10ppb, PE = 0.96, 95% CI = −0.10 to 2.02; 250ppb produced no larvae at all and were excluded from the analysis to aid model fit). However, at 10ppb, those colonies that did produce larvae produced significantly fewer than the control, which was not the case at 5ppb (Figure 2b, hurdle (positive‐count output), 5ppb, parameter estimate (PE) = 1.24, 95% CI = 0.16 to 2.32; 10ppb, PE = 0.96, 95% CI = −0.10 to 2.02).

**Figure 3:**
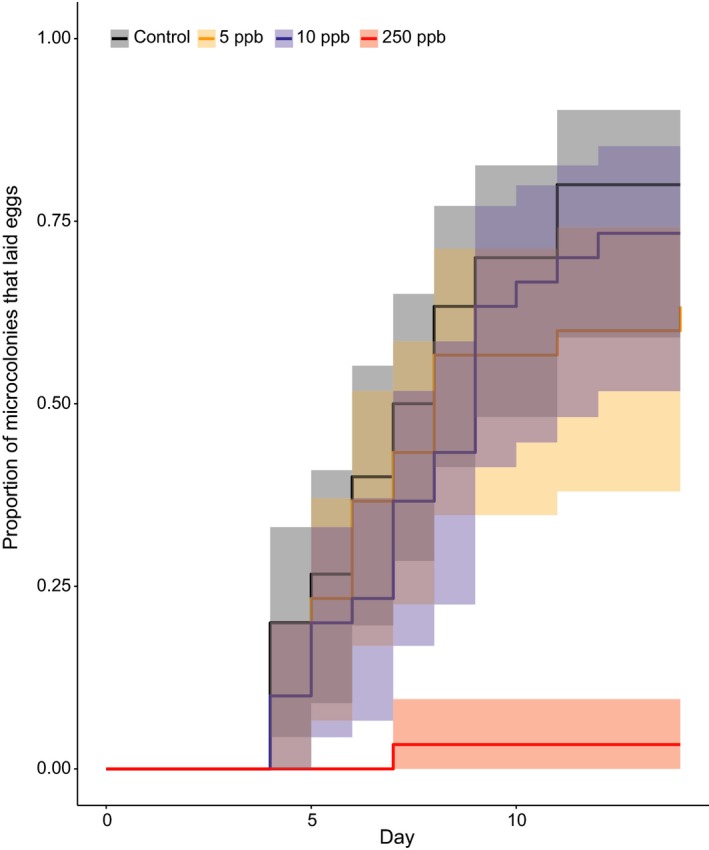
Kaplan–Meier plot depicting the proportion of microcolonies that had produced eggs by each day of the experiment. ppb = parts per billion of sulfoxaflor in sucrose

The latency to lay eggs (time to when a microcolony first laid eggs) did not differ between control groups and 5 and 10ppb treatment groups (Figure 3, coxme, 5ppb parameter estimate (PE) = −0.41, 95% confidence intervals (CI) = −1.02 to 0.19; 10ppb, PE = −0.45, 95% CI = −1.04 to 0.14; 250ppb, PE = −4.40, 95% CI = −6.42 to 2.38), suggesting that the speed at which ovaries developed, and eggs were laid, did not drive the observed differences in fecundity.

**Figure 4:**
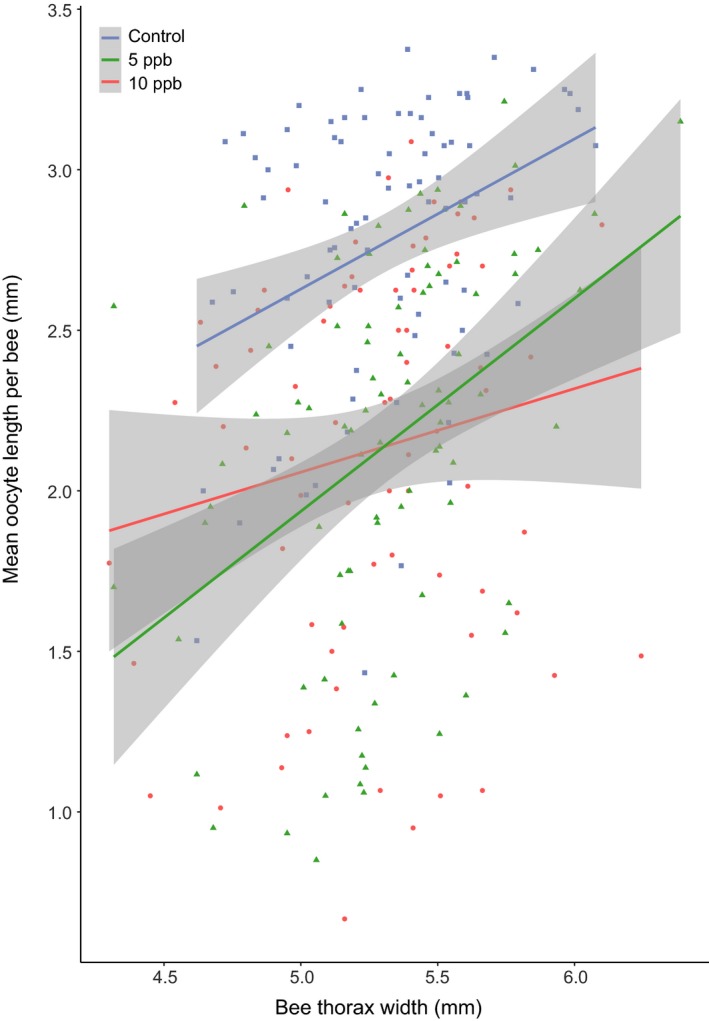
The mean oocyte length per bee plotted against bee thorax width (mm). Ppb = parts per billion of sulfoxaflor in sucrose

Of the 373 bees we dissected, 254 had developed ovaries (Control group: 83/102 (81%) developed: measured; 5ppb: 88/110 (80%); 10ppb: 76/105 (72%); 250ppb: 7/56 (12%)) with only the positive control differing significantly from the control group (Figure S1, glmer, binomial distribution, 5ppb, PE = 0.15, 95% CI = −0.72 to 1.02; 10ppb, PE = −0.42, 95% CI = −1.25 to 0.42; 250ppb, PE = −4.74, 95% CI = −6.13 to −3.34). Furthermore, we found no significant effect of sulfoxaflor exposure on mean or maximum oocyte size per bee (mean oocyte size per bee; Figure 4, lmer, 5ppb parameter estimate (PE) = −0.73, 95% CI = −1.81 to 0.36; 10ppb, PE = −0.42, 95% CI = −1.73 to 0.89; maximum oocyte size per bee; Figure S2, Table S2; lmer, 5ppb parameter estimate (PE) = −0.46, 95% CI = −1.29 to 0.37; 10ppb, PE = −0.31, 95% CI = −1.44 to 0.82). Despite no overall effect of sulfoxaflor exposure on mean oocyte size per bee at the 95% confidence level, the model containing both treatment and bee size was strongly supported (*wi* (treatment + bee size) = 0.847) with the null model, and models containing just treatment and bee size in isolation receiving no support (*wi* (treatment) = 0.00), (*wi* (bee size) = 0.00)), (*wi* (null model) = 0.00), (Table S1).

**Figure 5:**
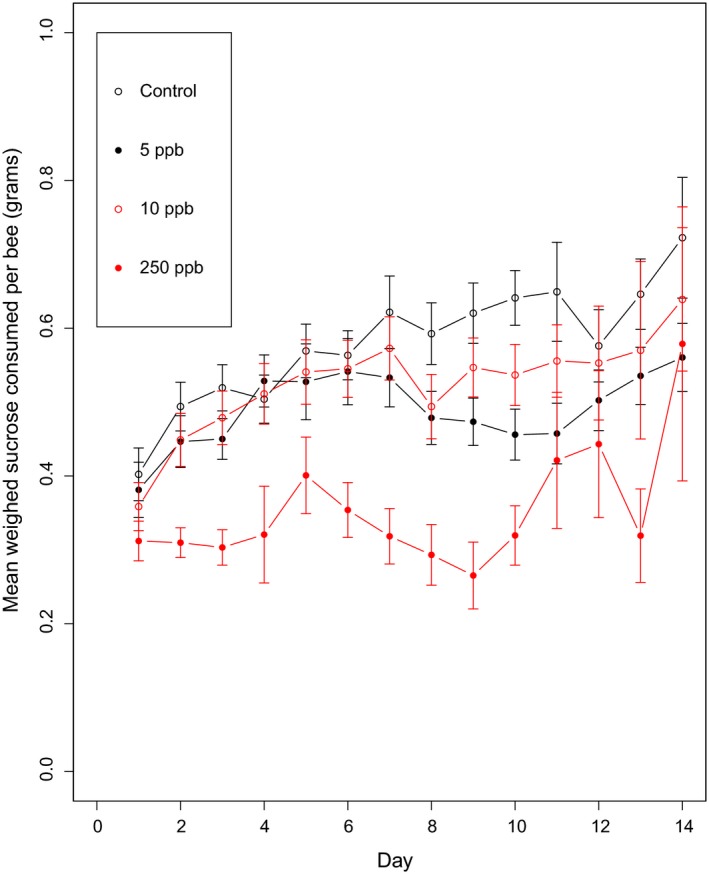
The mean (±*SE*) amount of sucrose consumed (grams) per bee. Ppb = parts per billion of sulfoxaflor in sucrose

Another potential driver of lower fecundity is a reduction in feeding, as a consequence of insecticide exposure (Laycock et al., 2012). Sulfoxaflor‐exposed microcolonies had reduced sucrose consumption per bee at 5 and 250ppb, but not significantly so at 10ppb (Figure 5, lmer, 5ppb, PE = −0.09, 95% CI = −0.17 to −0.02; 10ppb PE = −0.06, 95% CI = −0.13 to 0.02; 250ppb, PE = −0.23, 95% CI = −0.31 to −0.16).

We further found no effect of sulfoxaflor exposure on worker survival at 5 or 10ppb (Figure S3, coxme, 5ppb parameter estimate (PE) = −0.46, 95% confidence intervals (CI) = −1.44 to 0.52; 10ppb PE = 0.13, 95% CI = −0.75 to 1.01) but microcolonies exposed to 250ppb had fewer workers surviving throughout the experiment (Figure S3, coxme, 250ppb parameter estimate (PE) = 2.07, 95% confidence intervals (CI) = 1.35 to 2.79).

## 4 DISCUSSION

Our results showed that sulfoxaflor exposure can negatively impact bumblebee egg laying, at least for workers in a queenless environment, with subsequent consequences for the number of larvae found within microcolonies. Sulfoxaflor exposure also resulted in a reduction in sucrose consumption per bee, which could be a possible driver of the observed differences in egg laying. Ultimately our results confirm that sulfoxaflor exposure at the levels we tested could be hazardous to bumblebees and suggest that reduced egg laying is a possible mechanism driving previously described effects of sulfoxaflor exposure on bumblebee colony reproductive output (Siviter, Brown, et al., 2018).

We previously found that sulfoxaflor exposure, early in the colony life cycle, influences the reproductive output of bumblebee colonies, although the mechanism that drove these effects remained unknown as we found no impact of sulfoxaflor exposure on bee foraging, or, in subsequent experiments, bee cognitive performance (Siviter, Brown, et al., 2018; Siviter, Scott, et al., 2019). Here we show that sulfoxaflor exposure at 5ppb and 250ppb can influence the egg‐laying of worker bumblebees, although we found no evidence at the 95% confidence level to support the same effect at 10ppb. It is not clear whether the lack of effect at 10ppb reflects true biological differences or statistical power. Nevertheless, the sublethal consequences of insecticides are not always dose‐dependent (Samuelson et al., 2016), and so further research would be necessary to establish the true shape of this relationship. Ultimately, our study provides evidence for an effect of sulfoxaflor exposure on egg laying at 5ppb. If similar effects to those observed in this experiment occur when queen bumblebees are exposed to sulfoxaflor, this has the potential to drive previously observed differences in colony reproductive output (Siviter, Brown, et al., 2018).

Despite these effects on egg laying, we found no detectable effect of sulfoxaflor exposure at 5 and 10ppb on both the likelihood of bees showing evidence of ovarian development, nor on terminal oocyte size. Interestingly, Laycock et al. (2012) also demonstrated that imidacloprid exposure, despite not influencing ovarian development, reduced brood production, with the authors suggesting that the reduction in fecundity was driven by reduced feeding rates in exposed colonies. In insects, both carbohydrate intake (sucrose) and protein intake (pollen) are essential for brood development (Boggs, 1997; Laycock et al., 2012; Murphy, Launer, & Ehrlich, 1983; Rotheray, Osborne, & Goulson, 2017). In our experiment, egg laying in the pollen balls provided occurred in the first week of the experiment, so we were unable to determine whether sulfoxaflor exposure influenced pollen consumption. Our results did however show that sulfoxaflor‐exposed microcolonies consumed less sucrose than controls, suggesting that lower nutritional intake here could be a potential driver for the observed differences in egg laying.

Sulfoxaflor‐exposed microcolonies contained fewer larvae than control colonies. Impacts were evident at 5ppb on the likelihood of producing larvae, and at 10ppb on the number of larvae. During the experiment, we observed no evidence of dead or discarded larvae in any of the microcolonies, suggesting that reduced egg‐laying resulted in lower larval numbers, although we cannot rule out that sulfoxaflor exposure led to fewer eggs hatching. Sulfoxaflor exposure, however, could still influence larval growth and development and there are two alternative influencing factors that have yet to be explored: (a) the potential impact of sulfoxaflor exposure on brood care and (b) the direct impact of sulfoxaflor consumption on bumblebee larvae. In a recent experiment, Crall et al. (2018) demonstrated that bumblebee colonies exposed to the neonicotinoid imidacloprid have reduced nursing behaviour and thermoregulation, which could potentially impact larval development. Furthermore the nectar and pollen stores of both honeybees and bumblebees frequently contain numerous agrochemicals (Mitchell et al., 2017; Nicholls et al., 2018; Wu, Smart, Anelli, & Sheppard, 2012) and yet, despite evidence in honeybees suggesting that insecticide exposure can influence larval development (Wu et al., 2012), no studies to date have investigated the direct impact of insecticide exposure on bumblebee larval development. Bumblebee larvae have blind guts, and do not excrete waste material until pupation begins (Chapman, 1998), and thus while acute exposure might not have direct impacts on larval mortality or growth, chronic exposure over a prolonged period of time could result in bioaccumulation of insecticides, which could potentially influence larval mortality, development and emergence. Future research should focus on understanding the relationship between insecticide exposure and bumblebee larval development.

Bumblebee workers develop their ovaries when the founding queen is absent (Alaux et al., 2007; Amsalem et al., 2009) and microcolony‐based designs are therefore not a direct reflection of a healthy bumblebee colony. Typically, in cases when the queen is absent, one worker will dominate reproduction, and become a pseudo‐queen (Blacquière, Smagghe, Gestel, & Mommaerts, 2012). However, in contrast to our expectations, we found no evidence that one worker dominated, as several bees within each microcolony developed their ovaries. Without behavioural observations, or relating egg laying to individual workers, we cannot be sure whether one worker dominated the microcolonies or not. The microcolony dynamics, and in‐turn egg laying, are likely to be sensitive to the number of workers present (Babendreier, Reichhart, Romeis, & Bigler, 2008; Larrere & Couillaud, 1993; Laycock et al., 2012; Mommaerts et al., 2010). Having more workers within microcolonies could potentially increase egg laying, and reproduction, although it could also lead to greater competition (Reeve & Keller, 2001) and this would be less representative of a healthy colony. For microcolony‐based studies to become a ring‐tested methodology, the number of workers housed within microcolonies needs to be standardized. However, while reproduction in microcolonies obviously differs from bumblebee colony reproduction, our results demonstrate that they are a useful proxy for understanding the potential sublethal impacts of agrochemicals on bumblebees (Laycock et al., 2012).

Insecticide residue levels vary widely between exposure regimes, crops and application rates (Bonmatin et al., 2015) and currently there is a dearth of data on the likely residue levels of sulfoxaflor found in the nectar and pollen of treated crops, particularly at field realistic application rates. The best available contemporary residue data is largely based on post‐spray applications, applied during flowering (Cheng et al., 2018; United States Environmental Protection Agency, 2016; Xu et al., 2012), which is prohibited in Europe, dramatically reducing the residue levels that bees are likely to encounter (Centner, Brewer, & Leal, 2018). Spraying flowering crops is however not prohibited in many other geographical areas across the globe (Dow AgroSciences Australia Limited, 2018; Dow AgroSciences New Zealand, 2018; Dow AgroSciences South Africa, 2018; EPA, 2019), and additionally, even pre‐ or post‐bloom spraying could lead to direct spray of non‐target plants if their flowering period does not coincide with the target crop (e.g. wildflowers/weeds; particularly in orchard strips) (EFSA, 2013). Results in this experiment showed that chronic sulfoxaflor exposure can negatively influence the egg laying of bumblebees, confirming that sulfoxaflor can be hazardous to bumblebees. However, future studies should focus on understanding the potential risk that sulfoxaflor exposure poses and focus on generating sulfoxaflor residue data from a range of crops at field realistic application rates. A robust understanding of the residue levels of sulfoxaflor in various crops will allow regulators and policy‐makers to offer clear advice on mitigation (Centner et al., 2018) and legislation that can reduce the risk of sulfoxaflor exposure for pollinators.

Regulators are increasingly considering the potential impact of insecticides on wild bees such as bumblebees and solitary bees (Gradish et al., 2019; Sgolastra et al., 2019). Large‐scale field experiments (e.g. Rundlöf et al., 2015; Woodcock et al., 2017) are a means to detect sublethal effects of insecticide exposure on non‐target organisms and are vital for understanding the wider implications of pesticide use on wild pollinators, but they are often expensive and difficult to standardize across countries (Woodcock et al., 2017). When licencing insecticides for use, regulatory bodies such as EFSA use a tier‐based system (EFSA, 2013), whereby lower‐tiered studies that assess the direct mortality consequences of insecticide exposure are conducted to determine whether higher‐tier field‐realistic testing is needed. Tier 1 currently consist of LD50 and LC50 experiments that determine toxicity over 96 hr on honeybees, but these experiments do not consider (a) the consequences of chronic exposure, (b) the potential impact on non‐*Apis* bees and (c) the potential sublethal consequences of insecticide exposure (Gradish et al., 2019; Sanchez‐Bayo & Tennekes, 2017).

Our results here, along with others (Laycock et al., 2012), demonstrate that microcolony‐based studies can be used to assay the potential sublethal impacts of chronic insecticide exposure on bumblebees. Given that bumblebees are potentially more vulnerable to insecticide exposure than honeybees (Gradish et al., 2019; Rundlöf et al., 2015), it is vital that they (and other wild bees; Sgolastra et al., 2019) are represented in the regulatory process. We therefore recommend that regulatory bodies and policy‐makers consider using and developing microcolony‐based experiments for *Bombus* as standard within an ecotoxicology framework, alongside other ring‐tested methodologies. Failure to design and implement experiments that consider the sublethal impacts of novel insecticides on bumblebees, and other wild pollinators, will results in insecticides being licenced for use without a true understanding of the potential ecological consequences.

## AUTHORS' CONTRIBUTIONS

H.S., M.J.F.B. and E.L. conceived and designed the experiments. H.S. & J.H. collected the data. H.S. conducted the statistical analyses and wrote the first version of the manuscript. All authors contributed and approved subsequent drafts.

## Data Availability

Data available via the Dryad Digital Repository https://doi.org/10.5061/dryad.mkkwh70v8 (Siviter, Horner, Brown, & Leadbeater, 2019).
