# Supplementary material for: Sulfoxaflor exposure reduces egg laying in bumblebees Bombus terrestris
Source: J Appl Ecol. 2019 Oct 29;57(1):160–9. doi: 10.1111/1365-2664.13519 (PMC7004077; doi:10.1111/1365-2664.13519)
Supplement: Supplementary file 1 [file JPE-57-160-s001.docx]

**Sulfoxaflor exposure reduces bumblebee egg laying**

Harry Siviter^1†^, Jacob Horner^1†^, Mark J F Brown^1^ & Ellouise Leadbeater^1^

^1^School of Biological Sciences, Royal Holloway University of London, Egham, Surrey, TW20 0EX, UK

^†^These authors contributed equally

Corresponding author: Harry Siviter

School of Biological Sciences

Royal Holloway

University of London

Egham, Surrey, TW20 0EX, UK

Harry.Siviter.2016@live.rhul.ac.uk


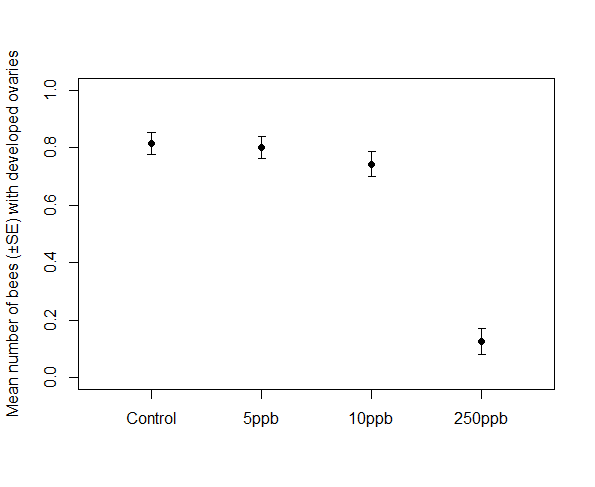


**Figure S1: The mean proportion (± SE) of bees with developed ovaries within each treatment group.**


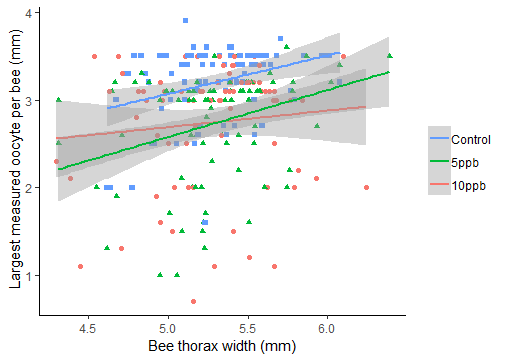


**Figure S2: The maximum oocyte** **length per bee plotted against bee thorax width.**


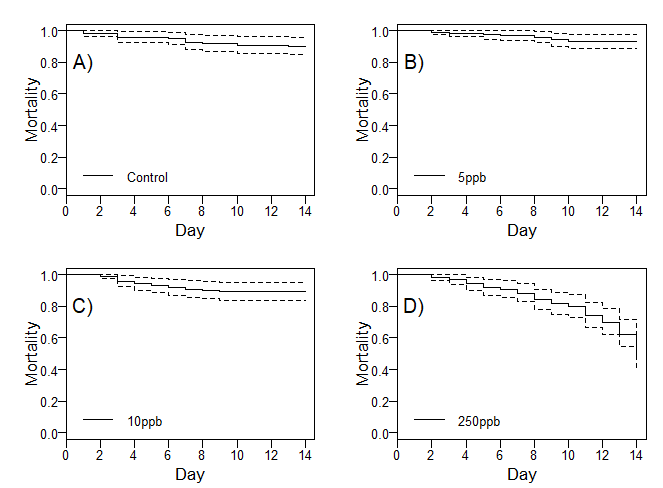


**Figure S3: Kaplan-Meier curves for indiviudal bee mortality in each treatment group (A = control, B = 5ppb, C = 10ppb, D = 250ppb).**
