# Supplementary material for: Sulfoxaflor exposure reduces egg laying in bumblebees Bombus terrestris
Source: J Appl Ecol. 2019 Oct 29;57(1):160–9. doi: 10.1111/1365-2664.13519 (PMC7004077; doi:10.1111/1365-2664.13519)
Supplement: Supplementary file 2 [file JPE-57-160-s002.pdf]

Table S1: Table of candiate models used for each analysis. Bold indicates that the model was selected within the anlaysis (wi ≥ 0.0 5)

**A) Latency to lay eggs: Mixed effect cox model (coxme). Colony of origin and microcolony included as random factors**	**AICc**	**ΔAICc**	***w* i**
**null model**	**509.10**	**0.00**	**1.00**
treatment	601.60	92.50	0.00
**B) Sucrose drank per bee (lmer): Colony of origin and microcolony included as random factors**	**AICc**	**ΔAICc**	***w* i**
**treatment + day**	**-1303.90**	**0.00**	**1.00**
treatment + day + treatment:day	-1279.50	24.40	0.00
day	-1275.50	28.33	0.00
treatment	-1250.70	53.16	0.00
null model	-1221.80	82.11	0.00
**C) Egg production (glmmTMB hurdle model): Colony of origin included as a random factor**	**AICc**	**ΔAICc**	***w* i**
**treatment**	**849.20**	**0.00**	**1.00**
null model	871.90	22.67	0.00
**D) Larvae production (glmmTMB hurdle model): Colony of origin included as a random factor**	**AICc**	**ΔAICc**	***w* i**
**treatment**	**439.90**	**0.00**	**0.94**
**null model**	**445.50**	**5.56**	**0.06**
**E) Ovary development (glmer, binomial distribution): Colony of origin and microcolony included as random factors**	**AICc**	**ΔAICc**	***w* i**
**treatment + bee size**	**323.20**	**0.00**	**1.00**
treatment	362.60	39.38	0.00
bee size	405.70	82.50	0.00
null model	436.50	113.32	0.00
**F) Mean oocyte size per bee (lmer): Colony of origin and microcolony included as random factors**	**AICc**	**ΔAICc**	***w* i**
**treatment + bee size**	**397.10**	**0.00**	**0.85**
**treatment + bee size + treatment:bee size**	**400.50**	**3.42**	**0.15**
treatment	414.70	17.55	0.00
bee size	443.20	46.11	0.00
null model	462.10	65.01	0.00
**G) Maximum ooyte size per bee (lmer) : Colony of origin and microcolony included as random factors**	**AICc**	**ΔAICc**	***w* i**
**treatment + bee size**	**443.30**	**0.00**	**0.89**
**treatment + bee size + treatment:bee size**	**447.80**	**4.52**	**0.09**
treatment	451.50	8.20	0.02
bee size	461.70	18.41	0.00
null model	470.20	26.93	0.00
**H) Worker survival (coxme) : Colony of origin and microcolony included as random factors**	**AICc**	**delta**	**weight**
**treatment**	**1034.30**	**0.00**	**1.00**
null model	1110.70	76.39	0.00
