# Supplementary material for: Sulfoxaflor exposure reduces egg laying in bumblebees Bombus terrestris
Source: J Appl Ecol. 2019 Oct 29;57(1):160–9. doi: 10.1111/1365-2664.13519 (PMC7004077; doi:10.1111/1365-2664.13519)
Supplement: Supplementary file 3 [file JPE-57-160-s003.pdf]

Table S2. Parameter estimates and 95% confidence intervals derived by model averaging across the confidence set of models. Parameters highlighted in bold have 95% confidence intervals that do not cross zero. Results correct to 2 decimal places.

**A) Latency to lay eggs**	**Estimate**	**Std. Error**	**Lower CI**	**Upper CI**
5ppb	-0.41	0.31	-1.02	0.19
10ppb	-0.45	0.30	-1.04	0.14
**250ppb**	**-4.40**	**1.03**	**-6.42**	**-2.38**
**B) Sucrose drank per bee**	**Estimate**	**Std. Error**	**Lower CI**	**Upper CI**
(Intercept)	0.49	0.04	0.41	0.57
**5ppb**	**-0.09**	**0.04**	**-0.17**	**-0.02**
10ppb	-0.06	0.04	-0.13	0.02
**250ppb**	**-0.23**	**0.04**	**-0.31**	**-0.16**
**day**	**0.01**	**0.00**	**0.01**	**0.02**
**C) Egg production**	**Estimate**	**Std. Error**	**Lower CI**	**Upper CI**
(Intercept)	2.92	0.06	2.80	3.04
**5ppb (count)**	**-0.16**	**0.08**	**-0.31**	**-0.01**
10ppb (count)	-0.10	0.07	-0.24	0.04
**250ppb (count)**	**-1.30**	**0.31**	**-1.91**	**-0.70**
(Intercept zero)	-1.53	0.77	-3.03	-0.03
5ppb (zero)	0.89	0.69	-0.45	2.24
10ppb(zero)	0.48	0.70	-0.89	1.84
**250ppb (zero)**	4.88	1.05	2.82	6.93
**D) Larvae production**	**Estimate**	**Std. Error**	**Lower CI**	**Upper CI**
(Intercept count)	2.00	0.16	1.68	2.31
5ppb (count)	0.04	0.13	-0.22	0.30
**10ppb(count)**	**-0.38**	**0.17**	**-0.69**	**-0.12**
(Intercept zero)	-0.69	0.39	-1.46	0.08
**5ppb (zero)**	**1.24**	**0.54**	**0.16**	**2.32**
10ppb(zero)	0.96	0.53	-0.10	2.02
**E) Ovary development**	**Estimate**	**Std. Error**	**Lower CI**	**Upper CI**
(Intercept)	-11.88	2.34	-16.47	-7.28
5ppb	0.15	0.44	-0.72	1.02
10ppb	-0.42	0.43	-1.25	0.42
**250ppb**	**-4.74**	**0.71**	**-6.14**	**-3.34**
**bee size**	**2.61**	**0.46**	**1.72**	**3.51**
**F) Mean oocyte size per bee**	**Estimate**	**Std. Error**	**Lower CI**	**Upper CI**
(Intercept)	0.31	0.59	-0.85	1.47
5ppb	-0.73	0.55	-1.81	0.36
10ppb	-0.42	0.67	-1.73	0.89
**bee size**	**0.46**	**0.11**	**0.24**	**0.67**
bee size:5ppb	0.02	0.10	-0.18	0.23
bee size:10ppb	-0.04	0.13	-0.28	0.21
**G) Maximum oocyte size per bee**	**Estimate**	**Std. Error**	**Lower CI**	**Upper CI**
(Intercept)	1.14	0.62	-0.09	2.36
5ppb	-0.46	0.42	-1.29	0.37
10ppb	-0.31	0.57	-1.44	0.82
**bee size**	**0.38**	**0.12**	**0.15**	**0.61**
bee size:5ppb	0.01	0.08	-0.15	0.16
bee size:10ppb	-0.02	0.11	-0.24	0.19
**H) Worker survival**	**Estimate**	**Std. Error**	**Lower CI**	**Upper CI**
5ppb	-0.46	0.50	-1.44	0.52
10ppb	0.13	0.45	-0.75	1.01
**250ppb**	**2.07**	**0.37**	**1.35**	**2.79**
